# A combination of genetically engineered oncolytic virus and melittin-CpG for cancer viro-chemo-immunotherapy

**DOI:** 10.1186/s12916-023-02901-y

**Published:** 2023-05-24

**Authors:** Amirhossein Bahreyni, Huitao Liu, Yasir Mohamud, Yuan Chao Xue, Yiyun Michelle Fan, Yizhuo Lyanne Zhang, Honglin Luo

**Affiliations:** 1 Centre for Heart Lung Innovation, St Paul’s Hospital, Vancouver, BC V6Z 1Y6 Canada; 2 Department of Pathology and Laboratory of Medicine, University of British Columbia, Vancouver, BC V6Z 1Y6 Canada; 3 Department of Experimental Medicine, University of British Columbia, Vancouver, BC V6Z 1Y6 Canada; 4 Department of Cellular and Physiological Sciences, University of British Columbia, Endowment Lands, Canada

**Keywords:** Virotherapy, Immunotherapy, Chemotherapy, Coxsackievirus B3, Melittin, Immunogenic cell death

## Abstract

**Background:**

Immunotherapy has emerged as an efficient therapeutic approach for cancer management. However, stimulation of host immune system against cancer cells often fails to achieve promising clinical outcomes mainly owing to the immunosuppressive characteristics of the tumor microenvironment (TME). Combination therapeutics that can trigger sustained immunogenic cell death (ICD) have provided new opportunities for cancer treatment.

**Methods:**

In this study, we designed and applied an ICD inducer regimen, including a genetically engineered oncolytic virus (miRNA-modified coxsackieviruses B3, miR-CVB3), a pore-forming lytic peptide (melittin, found in bee venom), and a synthetic toll-like receptor 9 ligand (CpG oligodeoxynucleotides), for breast cancer and melanoma treatment. We compared the anti-tumor efficacy of miR-CVB3 and CpG-melittin (CpGMel) alone and in combination (miR-CVB3 + CpGMel) and investigated possible mechanisms involved.

**Results:**

We demonstrated that miR-CVB3 + CpGMel had no major impact on viral growth, while enhancing the cellular uptake of CpGMel in vitro. We further showed that combination therapy led to significant increases in tumor cell death and release of damage-associated molecular patterns compared with individual treatment. In vivo studies in 4T1 tumor-bearing Balb/c mice revealed that both primary and distant tumors were significantly suppressed, and the survival rate was significantly prolonged after administration of miR-CVB3 + CpGMel compared with single treatment. This anti-tumor effect was accompanied by increased ICD and immune cell infiltration into the TME. Safety analysis showed no significant pathological abnormalities in Balb/c mice. Furthermore, the developed therapeutic regimen also demonstrated a great anti-tumor activity in B16F10 melanoma tumor-bearing C57BL/6 J mice.

**Conclusions:**

Overall, our findings indicate that although single treatment using miR-CVB3 or CpGMel can efficiently delay tumor growth, combining oncolytic virus-based therapy can generate even stronger anti-tumor immunity, leading to a greater reduction in tumor size.

**Supplementary Information:**

The online version contains supplementary material available at 10.1186/s12916-023-02901-y.

## Background

Recently, cancer immunotherapy has gained considerable attention for management of various malignancies. However, the existence of an immunosuppressive tumor microenvironment (TME) in some tumors has become a major obstacle to achieve satisfying outcomes and often limits its clinical application. There is growing evidence suggesting that applying therapeutic agents with the capability of arousing anti-tumor immune responses through induction of immunogenic cell death (ICD) of tumor cells is an effective strategy to remodel the immunosuppressive TME [1, 2]. Induction of ICD can result in the release of tumor-associated antigens (TAAs) and danger-associated molecular patterns (DAMPs), followed by commencing anti-tumor immune response [3]. Exposure of calreticulin (CRT) on the surface of tumor cells together with the release of high-mobility group box 1 (HMGB1) and secretion of adenosine triphosphate (ATP) are considered the main markers for ICD [4]. Exposed CRT serves as an “eat me” signal that improves immunogenicity of the tumors [5], while released ATP acts as a “find me” signal to enhance immune cell infiltration into the TME [6]. Finally, extracellular release of HMGB1 induces inflammation to recruit additional immune cells [7]. Release of all these molecules (i.e., DAMPs) along with TAAs can stimulate antigen-presenting cells (macrophages and dendritic cells), leading to the activation of cytotoxic T lymphocytes toward cancer cells [8]. Hence, combining current cancer immunotherapeutic strategy with ICD inducers can repress tumor growth in a collaborative mechanism.

One of the strategies in cancer immunotherapy is oncolytic virotherapy [9]. Oncolytic viruses (OVs) are replication-competent viruses that are able to selectively target and replicate in tumor cells [10]. The effect of oncolytic virotherapy on cancer cells was originally thought to be direct lysis of infected cells. However, growing evidence suggests that treatment of tumor cells with OVs can induce a highly inflammatory TME and initiate an immune response against tumor cells [11, 12]. Release of TAAs, pathogen-associated molecular patterns (PAMPs), and DAMPs, along with OV-triggered production of diverse cytokines, are considered the main sources of OV-associated induction of anti-tumor immunity [13, 14]. Among diverse OVs that are being developed against different malignancies, coxsackievirus B3 (CVB3), a non-enveloped single-stranded RNA virus from the picornavirus family, has attracted attention owing to its superb oncolytic activity [15, 16]. It has been demonstrated that CVB3 is capable of infecting and destroying different tumor cells in vitro and in vivo including colon cancer, lung cancer, and breast cancer [17–19]. However, having said that, undesirable virus-induced side effects, including pancreotoxicity and cardiotoxicity, have been reported after applying it in vivo for the treatment of tumors [20]. Several efforts have been made to reduce CVB3-related toxicity. One effective strategy is to incorporate target sequences of organ-specific and/or tumor-suppressive microRNAs (miRNAs) into the virus genome, which has been proven to be effective in reducing CVB3-induced tissue toxicity [21–23]. In this scenario, upon internalization of miRNA-modified oncolytic virus into the cells that contain the specific miRNAs, the miRNAs will bind to their target sequences in the virus genome, resulting in the degradation of the viral mRNA [24].

Melittin is the major component of bee venom with 26 amino acid residues [25]. As a natural cationic peptide, it possesses numerous biological and pharmacological properties, such as modulating pro-inflammatory response, activating innate and adaptive immunity, and more importantly, stimulating tumor cell cytotoxicity [26, 27]. It has been shown that melittin is able to directly kill cancer cells through membrane permeability enhancement and consequent cell death [28, 29]. Due to its immunomodulatory and anti-tumor effects, melittin has been employed as a therapeutic agent against various cancers [30, 31]. It was demonstrated that oncolytic adenovirus carrying melittin gene showed promising anti-tumor efficacy in tumor-bearing mice [32]. Oligodeoxynucleotides (ODN) containing CpG motifs (CpG-sequence) is a well-known agonist for toll-like receptor 9 (TLR9) that can activate host defense mechanisms including induction of antigen-presenting cell maturation [33, 34]. Studies have shown that the combination of CpG sequences with oncolytic viruses can enhance the immune response against cancer cells, as compared to oncolytic therapy alone [35, 36].

In the current study, we aimed to combine different therapeutic strategies (i.e., CpG-melittin complex (CpGMel) and miR-CVB3) in order to achieve a potent anti-tumor response. The rationale of the proposed treatment is that both miR-CVB3 and melittin can directly lyse cancer cells and release TAAs into the TME, resulting in a more effective cancer treatment. Additionally, they are both inherent immunostimulatory agents, which can remodel the immunosuppressive TME when released together with TAAs, PAMPs, and DAMPs. Our result showed that the combination treatment of miR-CVB3 with CpGMel led to a significant enhancement in the rate of ICD in vitro and in vivo as compared to mono-treatment. Moreover, the proposed strategy was able to increase immune cell infiltration in the TME and impede tumor growth in both 4T1 and B16F10 tumor-bearing mice without causing significant toxicity.

## Methods

### Cell culture

The 4T1 cells (CRL-2539, a mouse triple-negative mammary tumor cell line isolated from Balb/c mice), MDA-MB231 cells (HTB-26™, a human triple-negative mammary tumor cell line), B16F10 cells (CRL-6475, a murine melanoma cell line isolated from C57BL/6 J mice), RAW 264.7 cells (TIB-71, macrophage-like cell line derived from Balb/c mice), and HeLa cells (CCL-2™, human cervical cancer cells) were purchased from the American Type Culture Collection. The 4T1, RAW 264.7, and B16F10 cells were cultured in Roswell Park Memorial Institute (RPMI) containing 10% FBS and 1% antibiotics (streptomycin, 100 μg/mL; penicillin, 100 U/mL). Hela cells and MDA-MB231 cells were maintained in Dulbecco’s modified Eagle’s medium (DMEM) containing 10% FBS and 1% antibiotics (streptomycin, 100 μg/mL; penicillin, 100 U/mL).

### Generation of recombinant CVB3

The miR-CVB3 was constructed as described previously [23]. In brief, 4 copies of miRNA-145 target sequence (TS), 4 copies of miRNA-216 TS, 2 copies of miRNA-1 TS, and 2 copies of miRNA-143 TS were inserted into the 5′untranslated region (UTR) of CVB3 genome. The resultant miR-CVB3 was propagated in Hela cells and kept at − 80 °C for further applications.

### Preparation and characterization of CpGMel

A fixed concentration (10 μg/ml) of CpG oligodeoxynucleotides (CpG ODNs, 1826, Integrated DNA Technologies) was added to increasing concentrations (0, 3, 6, 12, 25, 40 μg/ml) of melittin (> 85% in purity, M2272, Sigma-Aldrich) and the mixture was incubated at room temperature for 1 h. The formation of CpGMel complex was evaluated using gel retardation assay (2.5% agarose gel). Moreover, 2.5% agarose gel was applied to evaluate the probability of binding CpGMel to the surface of miR-CVB3 after incubating them at room temperature for 1 h and purifying miR-CVB3 using a centrifugal filter.

### Cellular uptake of miR-CVB3 and CpGMel

To evaluate the internalization/replication of miR-CVB3, 4T1 cells were seeded into the 8-well chamber slides (10^4^ cells per well) and 24-well plates (5 × 10^4^ cells per well). MDA-MB231 cells were also seeded into 24-well plates (5 × 10^4^ cells per well). The following day, cells were exposed to miR-CVB3 (multiplicity of infection (MOI) = 1), or miR-CVB3 + CpGMel (miR-CVB3 at an MOI of 1, melittin at a concentration of 10 μg/ml, and CpG ODNs at a dose of 5 μg/ml) for 1 h. Then, the media was removed and replaced with fresh media. For examination of viral internalization/replication by confocal microscopy, after additional 16-h incubation, cells were washed with phosphate-buffered saline (PBS). After fixation in 4% paraformaldehyde and permeabilization with 0.1% Triton X-100, cells were blocked with 3% bovine serum albumin (BSA) and then incubated with VP1 antibody (M47, Mediagnost, Germany) at 4 °C for overnight. Following additional incubation with Alexa Fluor® 488-conjugated secondary antibody (A11029, Invitrogen) at room temperature for 1 h, cells were washed with PBS, mounted with fluoroshield with 4, 6-diamidino-2-phenylindole (DAPI; F6057, Sigma-Aldrich), and subjected to Zeiss LSM 880 inverted confocal microscopy for imaging.

For measurement of viral entry/replication by western blotting, after additional 16-h incubation, both 4T1 and MDA-MB231 cells were lysed in buffer (10 mm HEPES pH 7.4, 50 mm Na pyrophosphate, 50 mm NaF, 50 mm NaCl, 5 mm EDTA, 5 mm EGTA, 100 μm Na3VO4, and 0.1% Triton X-100). Western blotting was conducted using VP1 antibody as previously described [23].

To evaluate the impact of miR-CVB3 on internalization of CpGMel, CpGMel was prepared using CpG(Cy5) (CpG ODNs, 1826, Integrated DNA Technologies). Cells were seeded and treated with CpG(Cy5), CpG(Cy5)Mel, or miR-CVB3 + CpG(Cy5)Mel (concentration of CpG(Cy5) for all treatments was 5 μg/ml) for 5 h. Confocal microscopy and flow cytometry (Gallios Flow Cytometer) were applied to investigate the uptake of CpG(Cy5). The results of flow cytometry were analyzed with FlowJo version 10 software.

### In vitro* anti-cancer study*

#### Cell viability assay

The 4T1 and MDA-MB231 cells were seeded onto a 96-well plate (10^4^ cells per well). The following day, cells were treated with miR-CVB3, CpGMel, or miR-CVB3 + CpGMel, as described above, for 24 and 48 h. Subsequently, 10 μl of MTS (3-(4,5-dimethylthiazol-2-yl)-5-(3-carboxymethoxyphenyl)-2-(4-sulfophenyl)-2H-tetrazolium) solution (G9243, Promega) was added into the culture, followed by another 3-h incubation. The absorbance of each solution was measured using a microplate reader (BioTek Synergy H1) at 490 nm. The OD values of untreated cells were set as 100% viability, and the percentage of inhibition was then calculated.

#### Apoptosis detection

The annexin V-Fluorescein isothiocyanate (FITC) staining was exploited to assess the apoptosis of cells treated with miR-CVB3, CpGMel, or miR-CVB3 + CpGMel. In brief, after exposure to miR-CVB3, CpGMel, or miR-CVB3 + CpGMel for 24 h, 4T1 cells were harvested and resuspended in annexin binding buffer (V13246, Thermofisher Scientific). Subsequently, annexin V-FITC (5 μl) (A13199, Thermofisher Scientific) reagent was introduced to each sample and incubated for 20 min in the dark. Finally, the stained cells were analyzed using flow cytometry. Data were analyzed with FlowJo version 10 software.

#### Detection of danger-associated molecular patterns (DAMPs)

Release and cell surface exposure of DAMPs, including ATP, CRT, and HMGB1, were analyzed after 4T1 cells were exposed to single treatment (miR-CVB3 or CpGMel) or combination therapy (miR-CVB3 + CpGMel) for 24 and 48 h. Specifically, the release of ATP into the supernatant was measured using RealTime-Glo™ extracellular ATP assay kit (GA5010, Promega) according to the manufacturer’s protocol. Briefly, 20 μl of 4 × RealTime-Glo™ extracellular ATP assay reagent was added into each culture. The luminescence was then measured using a microplate reader (BioTek Synergy H1). HMGB1 release was assessed by western blotting using HMGB1 primary antibody (651401, Biolegend). Briefly, the supernatant after treatment was collected and precipitated by the addition of equal volume of methanol and 0.25 volumes of chloroform. The mixture was vortexed and centrifuged for 10 min at 20,000 × g. The upper phase was discarded. Subsequently, 500 μl was added into interphase. Afterwards, the mixture was centrifuged for 10 min at 20,000 × g. Finally, protein pellet was dried at 55 °C, resuspended in protein loading buffer, and subjected to western blotting. For CRT detection, after treatment, cells were incubated for 60 min with anti-CRT (Alexa Fluor® 647) antibodies (ab196159, Abcam) at 4 °C in the dark. Following several washes, flow cytometry was applied to analyze the translocation of CRT. Confocal microscopy was also used to visualize CRT on the surface of the cells exposed to corresponding treatments. After fixation, the cells were incubated with anti-CRT (Alexa Fluor® 647) for 1 h, followed by DAPI straining.

#### Reverse transcription-quantitative polymerase chain reaction (RT-qPCR)

RT-qPCR was conducted to measure the gene level of TNF-α and IL-6 in 4T1 cells treated with CpGMel, miR-CVB3, and miR-CVB3 + CpGMel for 8 h. Primers for the RT-qPCR analysis were synthesized by Integrated DNA Technologies and presented in Table 1. Briefly, after incubation, total RNA was isolated using RNeasy Mini Kit (Qiagen, 74104, Qiagen). The qPCR reaction containing 1 μg of RNA was conducted applying the TaqMan™ RNA-to-CT™ 1-Step Kit (4392653, Thermo Fisher Scientific) on a ViiA 7 Real-Time PCR System (Applied Biosystems). The results were normalized to β-actin mRNA. Samples were run in triplicate and analyzed using comparative CT (2 − ΔΔCT) method with control samples and presented as relative fold changes.

**Table 1:** Designed primers for RT-qPCR

Target	Forward	Reverse
Murine *Tnfα*	5′-GTC CCC AAA GGG ATG AGA AGT T-3′	5′-GTT TGC TAC GAC GTG GGC TAC A-3′
Murine *Il6*	5′-ACA ACC ACG GCC TTC CCT AC-3″	5′-TCT CAT TTC CAC GAT TTC CCA G-3′
Murine *β-actin*	5′-CAT TGC TGA CAG GAT GCA GAA GG-3′	5′-TGC TGG AAG GTG GAC AGT GAG G-3′

#### Macrophage activation

To examine the activation of macrophages in vitro, the media of 4T1 cells treated with miR-CVB3, CpGMel, or miR-CVB3 + CpGMel for 12 h were transferred to the plates seeded with RAW 264.7 cells, followed by incubation for 24 h. Subsequently, RAW 264.7 cells were collected and stained with CD80-PE (B340153, Biolegend) and MHC-II-Alexa Fluor® 647 (B346505, Biolegend) antibodies for 30 min. The macrophage activation was then detected using a flow cytometer. Data were analyzed with FlowJo version 10 software.

### In vivo* anti-tumor study*

#### Animals

Six- to 8-week-old female Balb/c (000651, The Jackson Laboratory) and female C57BL/6 J (000664, The Jackson Laboratory) mice were used for the in vivo studies. All animal procedures were performed in compliance with strict guidelines for the care and use of laboratory animals and were approved by the Animal Care Committee at the University of British Columbia (A18-0275). The ARRIVE guidelines were used for reporting animal research [37].

#### Therapeutic effects in a murine breast cancer model

The 4T1 cells (5 × 10^5^ cells) in 100 μl of cold PBS were subcutaneously injected into the right flank of female Balb/c mice. After about 10 days, once tumor reached a palpable size (~ 50 mm^3^), 4T1 tumor-bearing Balb/c mice were randomly divided into 4 groups (*n* = 8 for each group), which were intratumorally treated with PBS, miR-CVB3 (10^5^ Plaque-Forming Unit (pfu)/mouse), CpGMel (CpG = 50 μg/mouse and melittin = 100 μg/mouse), or miR-CVB3 + CpGMel, respectively. Treatments were performed twice on days 0 and 5. The length and width of the tumors were measured every 3 days using a digital caliper, and tumor volumes were calculated using the formula of (volume = length × width^2^ × 0.52). Furthermore, the tumor suppression rate (TSR) was calculated using the following formula: TSR (%) = [1 − (tumor volume of the treated group)/(tumor volume of the control group)] × 100 (%). According to our approved protocol, humane endpoints were defined as follows: mice losing ≥ 20% of their initial body weight, observation of ulceration in ≥ 10% of the tumor region, the tumor size reaching ≥ 1.7 cm in diameter, or tumor weight exceeding 10% of body weight. Mice were kept for 40 days to evaluate the survival rate. Mice in each group were euthanized once they reached humane endpoints. Additionally, tumor metastasis into the lungs was assessed at the end of the experiment. Briefly, lung tissues were collected and fixed in 4% paraformaldehyde. Metastatic tumors in the lung were analyzed via hematoxylin–eosin (H&E) staining. Terminal deoxynucleotidyl transferase dUTP nick end labeling (TUNEL) assay was also performed on tumor tissues (collected at humane endpoints) to assess apoptosis according to the manufacturer’s protocol (G3250, Promega).

#### Safety analysis

To assess the safety of each treatment, the body weight of mice in each group was measured every 3 days until the experimental endpoint. For safety measurement, a different cohort of mice (*n* = 4 for each group) were treated with PBS, miR-CVB3, CpGMel, or miR-CVB3 + CpGMel as above. At 14 days post-treatment, mice were sacrificed. The heart, liver, spleen, lung, pancreas, and kidney were collected and fixed in 4% paraformaldehyde for H&E staining. In addition, facial blood was collected on day 14 for the blood biochemistry analysis for alanine aminotransferase (ALT), aspartate aminotransferase (AST), creatine (CREA), lipase (Lip), and cardiac troponin I levels by Advia 1800 (Advia 1).

#### Immune cell infiltration

Two weeks after treatment, mice were sacrificed, and tumor samples were paraffin-embedded and then sliced into 5 μm of thicknesses. The sections were deparaffinized, rehydrated, and then stained with anti-CD8 (sc-1177, Santa Cruz Biotechnology), anti-NK1.1 (14–5941-82, eBioscience), and anti-F4/80 (sc-377009, Santa Cruz Biotechnology) antibodies through immunohistochemistry (IHC), as previously described [38], using the MACH4 Universal HRP-Polymer Detection System (BRI4012H, Biocare Medical) and hematoxylin solution Gill II (GHS232, Sigma-Aldrich). Lastly, Aperio ScanScope AT (Digital slide scanner, Leica Biosystems Inc) was applied to attain whole-slide digital images. All the staining images were quantified using NIH ImageJ (version 1.52p) and the results were presented as relative optical density. Moreover, the level of IFN-γ, IL-6, and TNF-α, as well as translocated CRT and granzyme B, in tumor tissues was analyzed using immunofluorescence staining. Tumor tissues were fixed with 4% paraformaldehyde, permeabilized with 0.1% Triton X-100, blocked with 10% FBS, and incubated with target antibodies including anti-IFN-γ (505802, Biolegend), anti-IL-6 (504502, Biolegend), anti-TNF-α (506302, Biolegend), and anti-CRT antibodies overnight. The following day, tissues were stained with Alexa Fluor® 488-conjugated secondary antibody (A11029, Invitrogen) for 1 h, followed by mounting with DAPI. Zeiss LSM 880 inverted confocal microscopy was used to visualize proteins.

#### Inhibition of distant metastatic tumor

For the distant tumor model, at 4 days after transplanting 4T1 cells into the right flank of mice (primary tumor,* n* = 5), a distant tumor was implanted by subcutaneous injection of 4T1 cells (5 × 10^5^ cells) into the left flank of each mouse. The primary tumors were treated as described before. The length and width of the distant tumors were measured every three days using a digital caliper.

#### Therapeutic effects in a murine melanoma model

To evaluate anti-tumor activity of miR-CVB3 + CpGMel in melanoma tumor-bearing mice, B16F10 (5 × 10^5^ cells) cells in 100 μl of cold PBS were subcutaneously injected into the right flank of C57BL/6 J female mice. After 10 days, once the tumor reached a palpable size (~ 50 mm^3^), the mice were randomly divided into 4 groups (*n* = 3 for each group). Mice were then intratumorally treated with PBS, miR-CVB3, CpGMel, or miR-CVB3 + CpGMel, and tumor size was measured as described above. At the experimental endpoint, various mouse organs were harvested for H&E staining and tumor was collected for viral quantitation by immunostaining of viral capsid protein VP1. We also assessed the expression of the coxsackievirus and adenovirus receptor (CAR) in non-treated implanted tumors (n = 3 mice) by IHC using an anti-CAR antibody (A1822, ABclonal).

### Statistical analysis

Statistical analysis was conducted by GraphPad Prism V8.0.1 software and all data are expressed as mean ± standard deviation (SD) (*n* ≥ 3). The results were analyzed by unpaired Student’s *t* test or one-way ANOVA followed by Tukey’s test to determine differences. The differences between survival rates were assessed by log-rank test. *P*-value < 0.05 was considered to be statistically significant (*, *p* < 0.05; **, *p* < 0.01; ***, *p* < 0.001, ****, *p* < 0.0001).

## Results

### *Characterization of CpGMel and miR-CVB3* + *CpGMel*

**Fig. 1:**
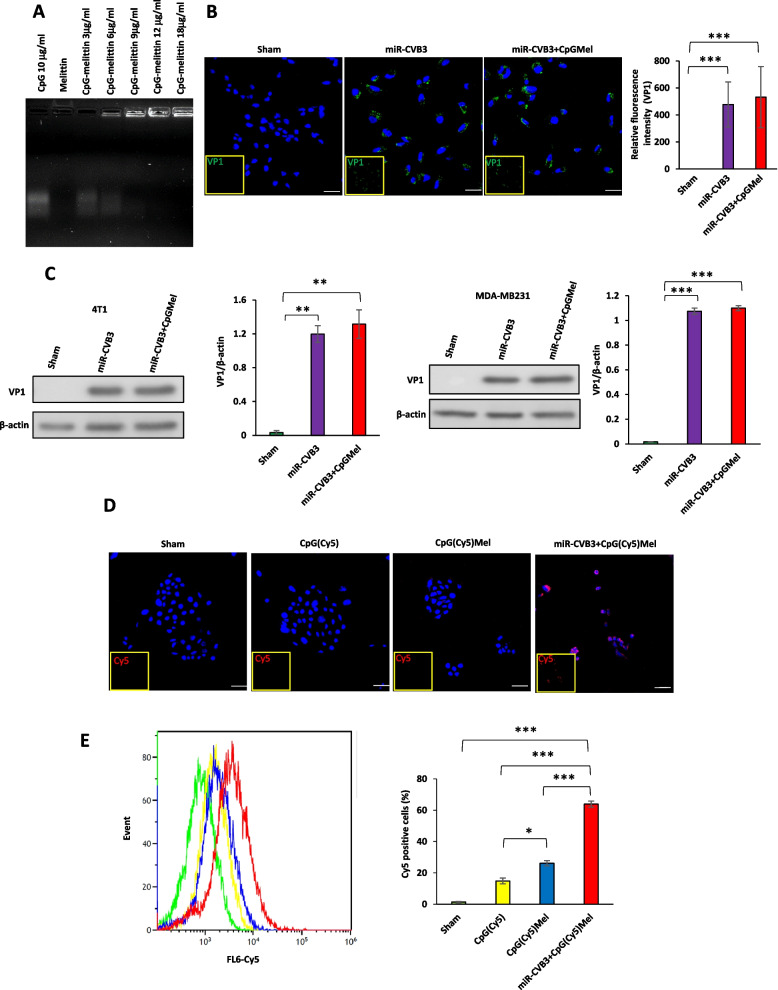
CpGMel does not affect miR-CVB3 internalization and replication in cancer cells, while miR-CVB3 enhances internalization of CpGMel to cancer cells. **A** Gel retardation assay was performed to verify proper formation of CpGMel. **B** VP1 expression in 4T1 cells after treatment with miR-CVB3 or miR-CVB3 + CpGMel for 16 h using confocal microscopy. The green and blue fluorescences represent VP1 and nucleus, respectively. Scale bar = 50 μm (left). The relative fluorescence intensity for each treatment was also quantified using NIH ImageJ and presented as mean ± SD (right). **C** Western blot analysis of VP1 level in 4T1 (left panels) or MDA-MB231 (right panels) cells treated as in **B**. Densitometric analysis of VP1 protein levels was conducted applying NIH ImageJ, normalized to β-actin. **D** Assessment of CpG(Cy5) internalization into 4T1 cells after 5-h treatment of cells with CpG(Cy5), CpG(Cy5)Mel, or miR-CVB3 + CpG(Cy5)Mel using confocal microscopy. The red and blue fluorescences represent Cy5 and nucleus, respectively. Scale bar = 50 μm. **E** Evaluation of CpG(Cy5) internalization into 4T1 cells after 5-h treatment of cells with CpG(Cy5), CpG(Cy5)Mel, or miR-CVB3 + CpG(Cy5)Mel using flow cytometry (*n* = 3–5). The concentrations of each agent for cell treatment were as follows: miR-CVB3 at an MOI of 1, melittin at a concentration of 10 μg/ml, and CpG ODNs at a dose of 5 μg/ml. Data in this figure were analyzed by one-way ANOVA followed by Tukey’s test to determine differences (*, *p* < 0.05; **, *p* < 0.01; ***, *p* < 0.001, ****, *p* < 0.0001)

Gel retardation assay was used to verify the formation of CpGMel and its binding to the surface of miR-CVB3. As shown in Fig. 1A, with an increase in the concentration of melittin, more CpG bound to the peptide, resulting in the complex staying at the top of the gel. Twelve micrograms per milliliter of melittin was considered the optimal concentration that could absorb 10 μg/ml of CpG ODNs. This was confirmed by the absence of a band corresponding to free CpG sequence, indicating that maximal binding of CpG to melittin occurred. Moreover, as demonstrated in Additional file 1: Fig. S1, no corresponding band for CpGMel was detected after filtration of solution containing CpGMel in the absence of miR-CVB3, while in the presence of miR-CVB3, the CpGMel complex was unable to pass through the filter and the CpGMel band was once again visible, suggesting an interaction between miR-CVB3 and CpGMel.

### Viral replication and uptake of CpG

The cellular internalization and replication of miR-CVB3 before and after introduction of CpGMel was examined by confocal microscopy (Fig. 1B) and western blotting (Fig. 1C). Compared with the administration of free miR-CVB3, no significant difference was observed in the expression of viral capsid protein VP1 in 4T1 cells and MDA-MB231 cells after applying miR-CVB3 + CpGMel, indicating that CpGMel does not appear to interfere with internalization and/or replication of miR-CVB3 in 4T1 cells. Moreover, the uptake of CpG ODNs was visualized and quantified by confocal microscopy (Fig. 1D) and flow cytometry (Fig. 1E), respectively. Our results showed that addition of melittin improved the internalization of CpG ODNs into the 4T1 cells, while treatment of cells with miR-CVB3 + CpGMel further enhanced CpG internalization as compared to melittin alone. Altogether, these results suggest that miR-CVB3 is able to internalize and replicate in 4T1 breast cancer cells in the presence of CpGMel, and that both melittin and miR-CVB3 can enhance cellular uptake of CpG ODNs.

### Cell viability and apoptosis induction

**Fig. 2:**
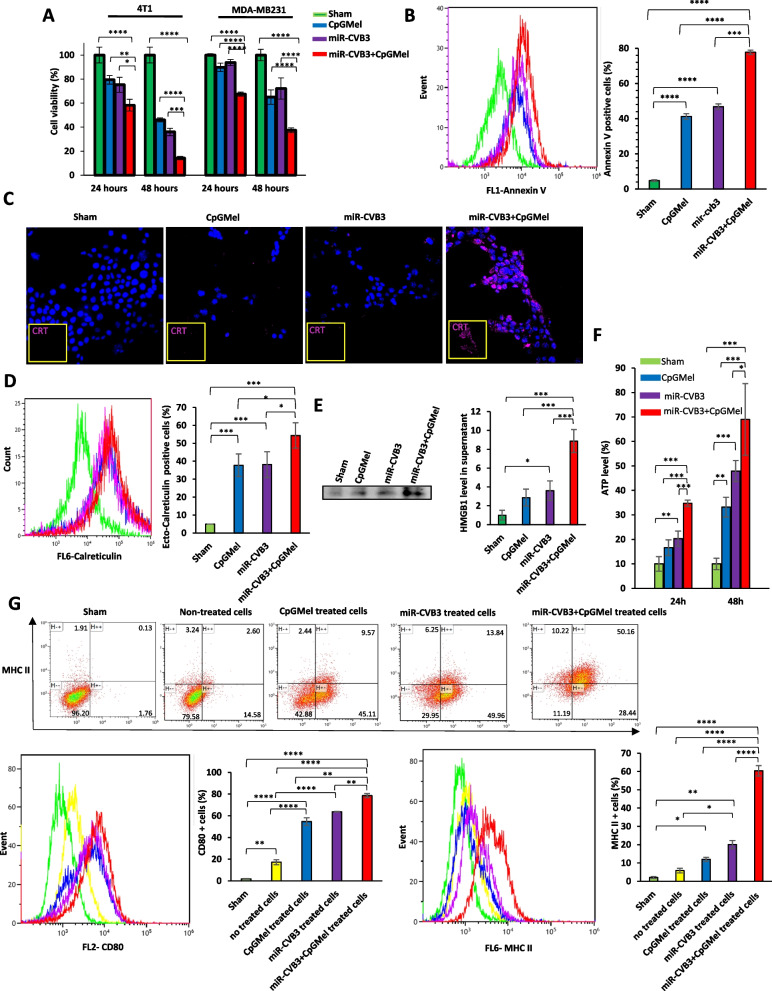
miR-CVB3 + CpGMel significantly increases apoptosis, DAMPs release, and macrophage activation compared with miR-CVB3 and CpGMel. **A** Cell viabilities of 4T1 and MDA-MB231 cells after 24- and 48-h incubation with CpGMel, miR-CVB3, or miR-CVB3 + CpGMel (*n* = 5). **B** 4T1 cells were analyzed for annexin-V after 24-h incubation with different therapeutic regimens as indicated (*n* = 3–5). **C** 4T1 cells were treated as above, followed by immunostaining of calreticulin. The purple and blue fluorescences represent CRT and nucleus, respectively. Scale bars = 50 μm. **D** Quantified calreticulin by flow cytometry (*n* = 3–5). **E** HMGB1 level in the supernatant of 4T1 tumor cells after various treatments were measured by western blotting (left) and quantified using NIH ImageJ (right). **F** Measurement of extracellular ATP at 24 and 48 h after indicated treatments (*n* = 5). The level of extracellular ATP in the different treatment groups was normalized to that of the sham group and presented the differences as %. **G** Evaluation of macrophage maturation (CD80 and MHC-II-positive cells) using flow cytometry after incubation of macrophages with supernatant collected from 4T1 cells treated with PBS (sham), CpGMel, miR-CVB3, and miR-CVB3 + CpGMel, and non-treated (*n* = 3). The concentrations of each agent for cell treatment were as follows: miR-CVB3 at an MOI of 1, melittin at a concentration of 10 μg/ml, and CpG ODNs at a dose of 5 μg/ml. Data in this figure were analyzed by one-way ANOVA followed by Tukey’s test to determine differences (*, *p* < 0.05; **, *p* < 0.01; ***,* p* < 0.001, ****, *p* < 0.0001)

After characterization of the miR-CVB3 + CpGMel, we then sought to determine its effects on cell viability and apoptosis. Firstly, cytotoxicity was quantifiably analyzed by MTS assay, after treatment of 4T1 and MDA-MB231 cells with PBS (sham), miR-CVB3, CpGMel, and miR-CVB3 + CpGMel for 24 or 48 h. Our results demonstrated that all three treatments were able to decline cell viability in a time-dependent manner, with miR-CVB + CpGMel group showing the highest cytotoxicity (Fig. 2A).

Apoptosis is a crucial mechanism underlying the anti-cancer activity of therapeutic agents. The average percentage of annexin V-staining positive cells (indicative of apoptotic cells) was 40% and 45% after treatment with CpGMel and miR-CVB3, respectively (Fig. 2B). However, combination therapy (miR-CVB3 + CpGMel) induced a significantly greater proportion of apoptotic cells (approximately 80%) in comparison with a single treatment (Fig. 2B). These results verify that combination therapy is more efficient to promote apoptosis in cancer cells.

### In vitro* induction of DAMP release and macrophage maturation*

It has been proven that ICD is able to generate an anti-tumor immune response in the TME [39]. In our next step, the induction of ICD by miR-CVB3 + CpGMel and single treatment was investigated by measuring DAMP release (i.e., CRT translocation to the surface of the cells, extracellular release of HMGB1 and ATP in treated cancer cells). While incubation with either miR-CVB3 or CpGMel resulted in a notable increase in CRT translation, the results obtained from confocal microscopy (Fig. 2C) and flow cytometry (Fig. 2D) revealed that treatment with miR-CVB3 + CpGMel induced significantly increased translocation of CRT in 4T1 cells exhibited significantly greater expression of CRT compared with either miR-CVB3 or CpGMel alone. Similarly, western blot analysis demonstrated that treatment of cells with miR-CVB3 + CpGMel resulted in a greater release of HMGB1 compared to treatment with miR-CVB3 and CpGMel alone (Fig. 2E). In addition, measurement of extracellular ATP after 24- and 48-h incubation of cells with each treatment revealed an increase in ATP level in a time-dependent manner (Fig. 2F). Among different treatments, the highest level of ATP in both time points was observed in the cells incubated with miR-CVB3 + CpGMel. Collectively, our data indicates that treatment with miR-CVB3 + CpGMel can more efficiently induce tumor cell release of DAMPs as compared to miR-CVB3 and CpGMel alone.

We next set out to test whether single or combination treatment can stimulate the immune system. We demonstrated that incubation of macrophages with supernatant collected from 4T1 cells treated with either miR-CVB3 or CpGMel induced moderate maturation of macrophages (13.84% and 9.57%, respectively), as evidenced by the induction of CD80 and MHC-II expression on macrophages (Fig. 2G). The cells positive for CD80 and MHC-II were significantly augmented to around 50% once they were cultured with the supernatant of the 4T1 cells treated with miR-CVB3 + CpGMel (Fig. 2G). Additionally, the level of pro-inflammatory cytokines (*Il-6* and *Tnf-α*) in 4T1 cells was analyzed by RT-qPCR after treating the cells with CpGMel, miR-CVB3, and miR-CVB3 + CpGMel. Administration of CpGMel showed no impact on RNA level of IL-6 while significantly increased RNA level of TNF-α was observed upon CpGMel treatment compared with sham-treated cells (Additional file 1: Fig. S2). Both miR-CVB3 and CVB3 + CpGMel treatments were able to enhance the RNA level of *Il-6* and *Tnf-α*; however, the highest induction of IL-6 and TNF-α was found after incubation of cells with miR-CVB3 + CpGMel (Additional file 1: Fig. S2). Overall, these findings indicate that miR-CVB3 + CpGMel could induce higher DAMP release, greater macrophage maturation, and enhanced pro-inflammatory cytokine production as compared to treatment with individual agent.

### In vivo* anti-tumor therapy and safety analysis*

**Fig. 3:**
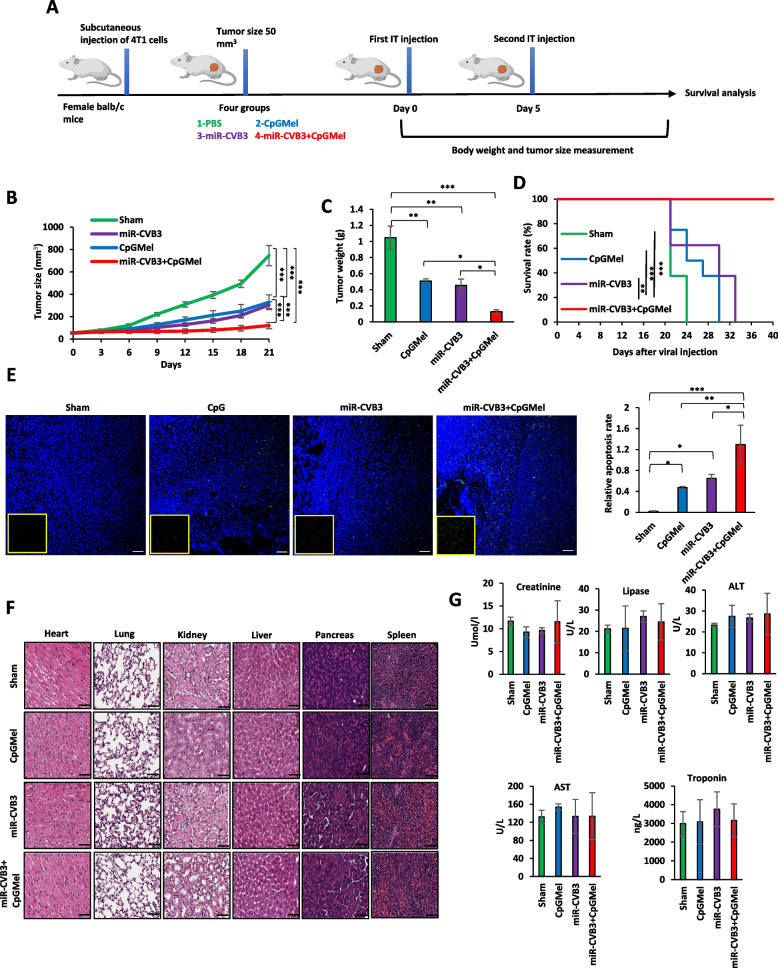
miR-CVB3 + CpGMel significantly inhibits tumor growth in 4T1 breast tumor-bearing Balb/c mice. **A** Diagram illustrating the therapeutic procedures. **B** The tumor size of mice in different groups (*n* = 8 mice/group). **C** Average tumor weights in different groups (*n* = 8 mice/group). **D** Animal survival curves following each treatment (*n* = 8 mice/group). **E** TUNEL staining of 4T1 tumor (left) and quantification of apoptosis rate by measuring fluorescent intensity signal (right, 3–5 slides/treatment). The green and blue fluorescences represent apoptotic cells and nucleus, respectively. Scale bar = 100 μm. **F** H&E staining of different tissues collected at day 14 post-treatment (representative of 3–5 slides/treatment). Scale bar = 50 μm. **G** Measurement of biochemical parameters, including creatinine, lipase, ALT, AST, and cardiac troponin I in the blood of 4T1 tumor-bearing mice after 21 days of treatment. Data represent the mean ± SD (*n* = 5). The concentrations of each agent for animal treatment were as follows: miR-CVB3 concentration at 10^5^ pfu/mouse, CpG concentration at 50 μg/mouse, and melittin concentration at 100 μg/mouse. Data in this figure were analyzed by one-way ANOVA followed by Tukey’s test to determine differences. The differences in survival rates were evaluated by log-rank test. *, *p* < 0.05; **, *p* < 0.01; ***, *p* < 0.001, ****,* p* < 0.0001. IT, intratumoral

After verification of the effectiveness in inducing cell death and immune activation in vitro, we then decided to assess the anti-tumor efficiency of the developed treatment in vivo in 4T1 breast tumor-bearing Balb/c mice. The anti-tumor therapeutic schedule is presented in Fig. 3A. When the implanted 4T1 tumors reached a volume of around 50 mm^3^, the mice were randomly separated into 4 groups, and each group received twice intratumoral injections on days 0 and 5. The tumor weight and size in each group are presented in Fig. 3B and Additional file 1: Fig. S3A, respectively. The data showed that both miR-CVB3 and CpGMel treatment were able to significantly delay tumor growth, resulting in an average tumor size of 303.25 mm^3^ and 329.26 mm^3^, respectively, at day 21, compared to 745.9 mm3 in the control group. However, the miR-CVB3 + CpGMel group exhibited the most outstanding anti-tumor performance, with an average tumor size of 120.75 mm^3^ at day 21 (Fig. 3B). The tumor suppression rate (TSR) was calculated in comparison to the PBS (sham) group. The TSR was 55.8% and 59.4% for the mice that received CpGMel and miR-CVB3, respectively, revealing that treatment with either CpGMel or miR-CVB3 alone efficiently suppressed 4T1 tumor growth (Additional file 1: Fig. S3B). Notably, the miR-CVB3 + CpGMel treatment displayed a more potent inhibition of tumor progression (83.8% TSR) than either treatment alone (Additional file 1: Fig. S3B). Results of tumor weight confirmed the ability of the miR-CVB3 + CpGMel therapy to suppress the growth of the malignant tumor (Fig. 3C). Moreover, Fig. 3D showed that treatment with miR-CVB3 + CpGMel significantly improved the survival rate of 4T1-bearing mice compared with the three other treatment groups. Furthermore, we found that administration of miR-CVB3 + CpGMel led to the highest apoptosis rate among all treatment groups, as measured by TUNEL staining of the tumor sections (Fig. 3E).

We also evaluated the safety profiles of the different treatments. As shown in Additional file 1: Fig. S3C, no prominent alteration in the body weight was observed following the various treatments. Histological analysis was also exploited to assess the safety of the major organs. H&E staining showed that all groups had normal histomorphology with no significant pathological abnormalities detected in the major organs (Fig. 3F). Furthermore, blood analyses of various biochemical markers revealed no evidence of cardiac, pancreatic, renal, and hepatic toxicity in any of the treatment groups (Fig. 3G).

### In vivo* assessment of inflammatory response and immune cell infiltration*

**Fig. 4:**
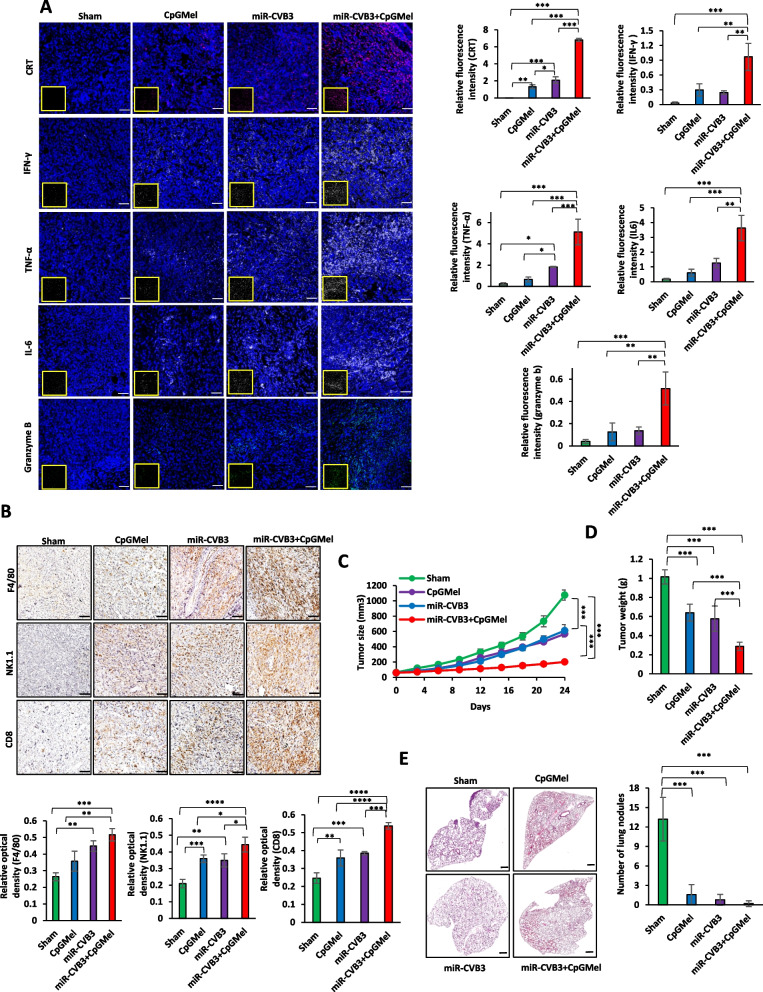
miR-CVB3 + CpGMel increases immune cell infiltration into the tumor microenvironment and reduces distant tumor growth and tumor metastasis into the lungs. **A** Immunostaining of CRT, IFN-γ, TNF-α, IL-6, and granzyme B in tumor tissues obtained at 14 days after indicated treatments. The purple, white, green, and blue fluorescences represent CRT, cytokines, granzyme B, and nucleus, respectively (left). Scale bar = 50 μm. The relative fluorescence intensity from 3 to 5 slides/treatment was quantified (right). **B** IHC staining of immune cell markers, F4/80, NK1.1, and CD8 in tumor tissues collected at day 14 post-treatment (top). Scale bar = 150 μm. Relative optical densities for immune cell markers are presented (bottom, *n* = 3–5 slides/treatment). **C**,** D** Average tumor size (untreated tumor) (**C**) and weight (**D**) of mice after various treatments (*n* = 5 mice for each group). **E** H&E staining of lungs harvested from mice treated with various formulations at the endpoint (left, *n* = 5 mice/group, scale bar = 500 μm), along with number of nodules in the lungs (right). The concentrations of each agent for animal treatment were as follows: miR-CVB3 at 10.^5^ pfu/mouse, CpG at 50 μg/mouse, and melittin at 100 μg/mouse. Data in this figure were analyzed by one-way ANOVA followed by Tukey’s test to determine differences (*, *p* < 0.05; **, *p* < 0.01; ***, *p* < 0.001, ****,* p* < 0.0001)

We next set out to further elucidate the anti-tumor mechanism of the developed treatments. The translocation of CRT in the TME was assessed by immunofluorescence (Fig. 4A). The tumor sections obtained from mice treated with miR-CVB3 + CpGMel presented the highest fluorescence intensity compared with the sham group and single therapy, verifying the distinguished potency of miR-CVB3 + CpGMel to induce ICD. It has been previously proven that TNF-α, IL-6, and IFN-γ play decisive roles in the response of immune cells against cancer cells [3]. Immunofluorescence was applied to assess the levels of TNF-α, IL-6, and IFN-γ in tumors. As expected, miR-CVB3 + CpGMel was the most effective treatment to boost the level of TNF-α, IL-6, and IFN-γ (Fig. 4A). Additionally, miR-CVB3 + CpGMel therapy resulted in a significant enhancement in the level of granzyme B in the TME compare to the sham group, suggesting higher cytotoxic effect of T cells (Fig. 4A).

We also analyzed the infiltration of macrophages, NK cells, and T cells into the TME with the IHC technique using anti-F4/80, NK1.1, and CD8 antibodies. We found that both CpGMel and miR-CVB3 were capable of increasing immune cells including F4/80-positive, NK1.1-positive, and CD8-positive cells in the TME, but the highest number of immune cells was observed after miR-CVB3 + CpGMel treatment (Fig. 4B). These results indicate that the combination treatment modality is the most efficient in stimulating immune cells against cancer cells.

### Growth inhibition of distant and metastatic tumor

We further investigated the effects of these treatment regimens on distant and pulmonary metastatic tumor. As shown in Fig. 4C, intratumoral injection of each treatment directly into primary, implanted tumors was able to impede the growth of distant, implanted tumors. At the end of the experiment, the average distant tumor sizes of mice that received CpGMel and miR-CVB3 were 568.8 and 610.9 mm^3^, while in the group treated with miR-CVB3 + CpGMel, the tumor size was 202.2 mm^3^. The most inhibitory effect, as measured by TSR (~ 64%), was observed in mice treated with miR-CVB3 + CpGMel (Additional file 1: Fig. S4). Consistently, results on tumor weight substantiated the ability of miR-CVB3 + CpGMel to suppress the growth of distant tumors (Fig. 4D). In addition, all treatments were able to prevent tumor metastasis into the lung compared with the sham group, as verified by H&E staining followed by counting the number of lung nodules (Fig. 4E). These findings support an immunotherapeutic effect of the developed treatment.

### Universal effect of the prepared treatment

**Fig. 5:**
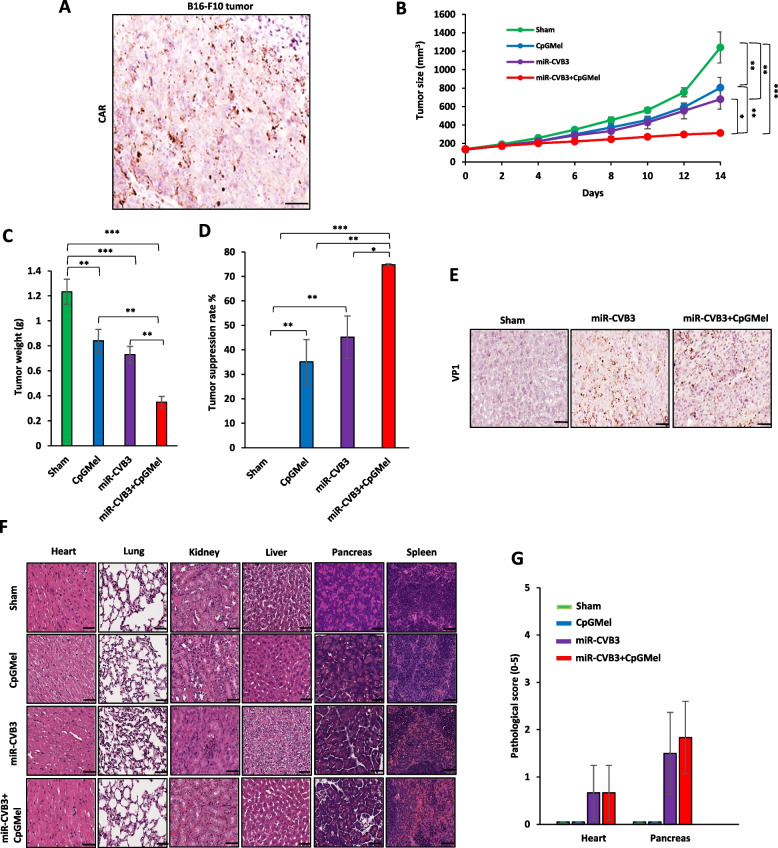
miR-CVB3 + CpGMel suppresses tumor growth in B16F10 tumor-bearing mice. **A** IHC staining of CAR in B16F10 tumor (representative of 3 slides). Scale bar = 100 μm. **B** The tumor size after different treatments (*n* = 3 mice/group). **C**, **D** Average tumor weights (**C**) and percentage of tumor suppression rate (**D**) in different groups (*n* = 3 mice/group). **E** IHC staining of VP1 in B16F10 tumor collected at day 14 post-treatment (representative of 2–3 slides/treatment). Scale bar = 100 μm. **F** H&E staining of different tissues collected at day 14 post-treatment (representative of 3 slides/treatment). Scale bar = 50 μm. **G** Pathological scores of the H&E staining (*n* = 3 mice/group). The concentrations of each agent for animal treatment were as follows: miR-CVB3 at 10.^5^ pfu/mouse, CpG at 50 μg/mouse, and melittin at 100 μg/mouse. Data in this figure were analyzed by one-way ANOVA followed by Tukey’s test to determine differences (*, *p* < 0.05; **, *p* < 0.01; ***, *p* < 0.001, ****, *p* < 0.0001)

We next tested the anti-tumor activity of these regimens in other mouse tumor models. Here we selected the B16F10-derived melanoma C57BL/6 J mouse model as one of the most immunologic malignancies [40]. We first evaluated and confirmed the expression of CAR, the primary receptor for CVB3 entry [15], in the TME of in B16F10 tumor-bearing mice (Fig. 5A). We then examined the therapeutic efficacy of miR-CVB3 + CpGMel along with single treatment in B16F10 tumor-bearing mice. We showed that administration of miR-CVB3 + CpGMel resulted in significantly reduced tumor size and weight compared with sham, CpGMel, or miR-CVB3 treatment (Fig. 5B and C). The TSR for the mice that received miR-CVB3 + CpGMel was 74.9%, significantly higher than that for mice that received CpGMel (35%) or miR-CVB3 (45%), indicating great therapeutic potency of the miR-CVB3 + CpGMel (Fig. 5D). Moreover, VP1 immunostaining revealed productive viral replication in tumor tissues when treated with miR-CVB3 or miR-CVB3 + CpGMel (Fig. 5E). No significant changes in the body weight of mice were observed after all treatments (Additional file 1: Fig. S5). However, minor toxicity, particularly pancreotoxicity and cardiotoxicity, was detected in mice treated with either miR-CVB3 or miR-CVB3 + CpGMel (Fig. 5F and G). Together, our data demonstrated the potency of the combined therapy in a different tumor model system.

## Discussion

In recent years, combination therapy has shown promising results for the suppression of various malignancies. Lately, employing OVs with the ability to lyse the cancer cells directly and modulate anti-tumor immunity has recaptured enhanced momentum in cancer treatment. For the current study, we applied a genetically engineered CVB3 through adding the target sequences of miRNAs that are overexpressed in normal tissues (miR-1 and miR-216) or downregulated in cancer cells (miR-143 and miR-145). It was previously shown that this miR-modified virus has a higher safety profile compared with wild type (WT)-CVB3 while maintaining the oncolytic activity [15]. Here we demonstrated that the combination of miR-CVB3 with CpGMel greatly improves the cytotoxic effect toward tumor cells both in vitro and in vivo as compared to the use of these agents separately.

To prepare the combination treatment, melittin was initially incubated with and bound to the CpG ODNs via electrostatic interaction to form CpGMel complex, followed by the addition of miR-CVB3. We found that binding CpG ODNs to melittin prevents the degradation of CpG ODNs by nucleases (data not shown), which is a significant challenge when using CpG ODNs alone for in vivo studies. Moreover, as CpG ODNs bind to melittin via electrostatic interaction, it is expected that alteration in pH, which is typically observed in the tumor microenvironment, leads to the separation of CpG ODNs from melittin without significantly affecting their functions. We confirmed that conjugation of CpGMel to miR-CVB3 has no major impact on viral fitness. Surface modification of Ovs has been widely applied for various purposes, including improving tumor targeting [41], preventing recognition and elimination by the immune system [42], and increasing therapeutic efficiency [43]. Berry et al. [43] demonstrated that conjugation of doxorubicin to the surface of reovirus does not influence virus performance, but rather elevates its oncolytic capacity. In this study, we also found that miR-CVB3 improves the cellular uptake of CpGMel. No specific receptors on the surface of the cells were identified for CpG entry. Therefore, it is possible that binding of miR-CVB3 to its receptor facilitates the internalization of CpGMels that are in close contact with miR-CVB3.

In this study, we assessed the oncolytic activity and cytotoxicity of miR-CVB3 as single treatment or combining with CpGMel(miR-CVB3 + CpGMel). We also investigated possible mechanism of cell death and anti-tumor immunity using in vitro and in vivo models. We found that both miR-CVB3 and melittin have the capability of direct lysis of cancer cells and inducing apoptosis. It is known that miR-CVB3 can replicate and rupture the infected cells [15, 21], and melittin kills the cells by creating pores in the plasma membrane [44]. Similar to our findings, different studies validated that the combination of Ovs with other common therapeutic approaches, such as chemotherapy and radiotherapy, could have synergistic effects and enhance cytotoxicity against cancer cells [45, 46].

In addition to direct lysis, both Ovs and melittin have been previously shown to elicit anti-tumor immunity [47, 48]. By rupturing cancer cells upon OV and melittin treatment, TAAs, DAMPs, and PAMPs are released into the TME, stimulating recruitment and activation of immune cells. Interestingly, we found that administrations of miR-CVB3 + CpGMel could cause ICD, as evidenced by the release of DAMPs, such as ATP and HMGB1, and increased translocation of CRT. All these DAMPs can be recognized by antigen-presenting cells (macrophages and dendritic cells), followed by T cell recruitment into the tumor [49, 50]. It is important to mention that in addition to the release of TAAs and DAMPs, miR-CVB3 + CpGMel has two inherent immunostimulatory agents, miR-CVB3 and CpG ODNs, which serve as PAMPs and work together with the released TAAs and DAMPs to initiate a robust immunity toward cancer cells. Our in vitro studies showed strong activation of macrophages after a combination treatment, while in vivo investigation revealed considerable infiltration of macrophages, T cells and NK cells into the TME. Combination therapy leads to a significant rise in the level of proinflammatory cytokines including TNF-α and IL-6 both in vitro and in vivo, which play a crucial role in the induction of immune response. Accumulating evidence suggests that local treatment of established tumors with ICD inducer can result in remission of distant tumors, signifying the establishment of systemic immunity [51]. Here we also showed that local administration of miR-CVB3 + CpGMel can effectively delay the progression of distant tumors, suggesting a strong capacity of the developed treatment to induce systemic immunity against tumor cells. Lastly, our investigation showed that CAR is expressed in B16F10-derived tumors and treatment with miR-CVB3 + CpGMel markedly represses tumor progression in B16F10 tumor-bearing mice. Although miR-CVB3 + CpGMel showed significant anti-tumor efficacy with an appropriate safety profile in both tumor-bearing Balb/c and C57BL/6 J mice, our results indicate that administration of the treatment led to a higher pathological score in C57BL/6 J mice compared to Balb/c mice. One possible explanation for this discrepancy is the different levels of miRNAs in distinct tissues of the two strains, leading to varying degrees of virus replication and subsequent toxicity. However, more in-depth investigations are needed to gain a better understanding of how each strain responds to miR-CVB3 infection. Such investigations will be crucial in optimizing the use of this treatment and ensuring its safety and efficacy in a broader range of contexts.

## Conclusions

In this study, the benefit of a combination therapy has been demonstrated using miR-CVB3, melittin, and CpG ODNs. It was shown that miR-CVB3 can improve the internalization of CpGMel into cancer cells, whereas addition of CpGMel does not affect virus performance. Our results proved that the combination therapy (miR-CVB3 + CpGMel) elicits greater tumor ICD in vitro and in vivo compared to individual treatment (miR-CVB3 and CpGMel). The release of DAMPs as a result of ICD along with miR-CVB3 and CpG ODNs induce increased recruitment of immune cells in the TME and initiate anti-tumor antigen-specific T cell response. Significant tumor suppression was achieved after applying miR-CVB3 and CpGMel as the single treatment; however, additive effect was observed following the administration of miR-CVB3 + CpGMel. These findings verify that the combination of cancer immunotherapy, which is based on OV, with a chemotherapeutic agent can be a potential strategy for further clinical applications.

## Supplementary Information

**Additional file 1:** **Fig S1.** Examination of possible interaction between CpGMel and the surface of miR-CVB3. Fig S2. qRT-PCR result of the level of TNF-α and IL-6 RNA in 4T1 cells. Fig S3.Tumor volume curves for individual mouse after indicated treatment.Tumor suppression rate after different treatments.Changes in body weights of mice after various treatment. Fig S4. Suppression rates of different treatments against the distant tumor. Fig S5. Body weights of C57BL/6 J mice after different treatments

**Additional file 2.**

## Data Availability

All data analyzed during this study are included in this published article and additional file.

## Abbreviations

ALT: Alanine aminotransferase
AST: Aspartate aminotransferase
ATP: Adenosine triphosphate
CAR: Coxsackievirus and adenovirus receptor
CpGMel: CpG-melittin complex
CREA: Creatine
CRT: Calreticulin
CVB3: Coxsackievirus B3
DAMPs: Danger-assiciated molecular patterns
DAPI: 4, 6-Diamidino-2-phenylindole
FITC: Fluorescein isothiocyanate
HMGB1: High-mobility group box 1
ICD: Immunogenic cell death
IHC: Immunohistochemistry
Lip: Lipase
miRNAs: MicroRNAs
MOI: Multiplicity of infection
MTS: 3-(4,5-Dimethylthiazol-2-yl)-5-(3-carboxymethoxyphenyl)-2-(4-sulfophenyl)-2H-tetrazolium
ODNs: Oligodeoxynucleotides
OVs: Oncolytic viruses
PAMPs: Pathogen-associated molecular patterns
PBS: Phosphate-buffered saline
RT-qPCR: Reverse transcription-quantitative polymerase chain reaction
TAAs: Tumor-associated antigens
TME: Tumor microenvironment
TS: Target sequence
TSR: Tumor suppression rate
TUNEL: Terminal deoxynucleotidyl transferase dUTP nick end labeling
UTR: Untranslated region
