# Supplementary material for: A combination of genetically engineered oncolytic virus and melittin-CpG for cancer viro-chemo-immunotherapy
Source: BMC Med. 2023 May 24;21:193. doi: 10.1186/s12916-023-02901-y (PMC10210435; doi:10.1186/s12916-023-02901-y)
Supplement: Supplementary file 1 — Additional file 1: Fig S1. Examination of possible interaction between CpGMel and the surface of miR-CVB3. Fig S2. qRT-PCR result of the level of TNF-α and IL-6 RNA in 4T1 cells. Fig S3.Tumor volume curves for individual mouse after indicated treatment.Tumor suppression rate after different treatments.Changes in body weights of mice after various treatment. Fig S4. Suppression rates of different treatments against the distant tumor. Fig S5. Body weights of C57BL/6 J mice after different treatments [file 12916_2023_2901_MOESM1_ESM.pptx]

## Slide 1
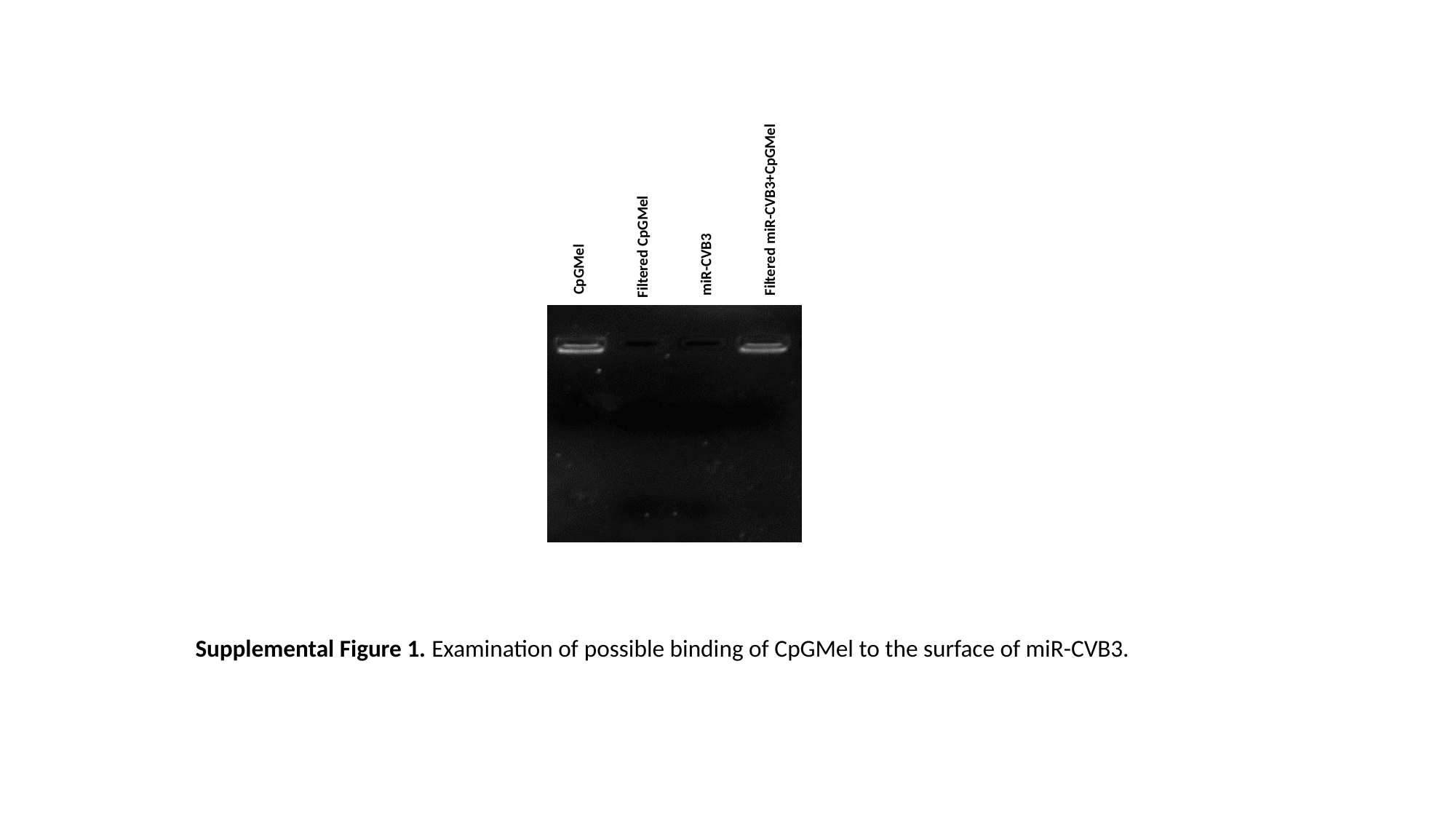

Filtered miR-CVB3+CpGMel
Filtered CpGMel
miR-CVB3
CpGMel
Supplemental Figure 1. Examination of possible binding of CpGMel to the surface of miR-CVB3.

## Slide 2
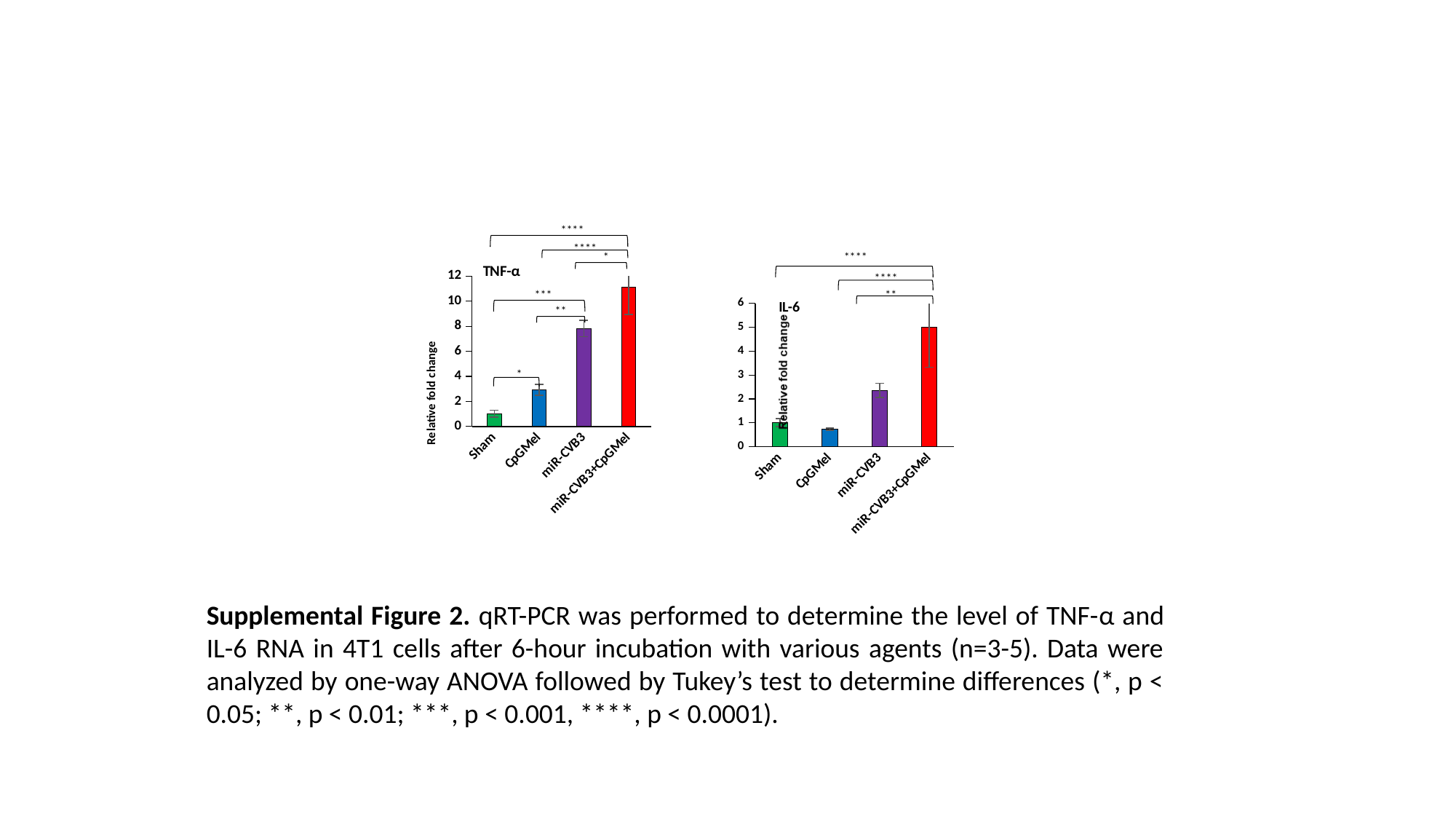

****
****
*
### Chart: TNF-α
| Category | |
|---|---|
| Sham | 1.0180615040591054 |
| CpGMel | 2.922843560373193 |
| miR-CVB3 | 7.80763530634373 |
| miR-CVB3+CpGMel | 11.100806416264899 |***
**
*
****
****
**
### Chart: IL-6
| Category | |
|---|---|
| Sham | 1.0112604835005756 |
| CpGMel | 0.7389455800301891 |
| miR-CVB3 | 2.352668733253656 |
| miR-CVB3+CpGMel | 4.99560987441005 |Supplemental Figure 2. qRT-PCR was performed to determine the level of TNF-α and IL-6 RNA in 4T1 cells after 6-hour incubation with various agents (n=3-5). Data were analyzed by one-way ANOVA followed by Tukey’s test to determine differences (*, p < 0.05; **, p < 0.01; ***, p < 0.001, ****, p < 0.0001).

## Slide 3
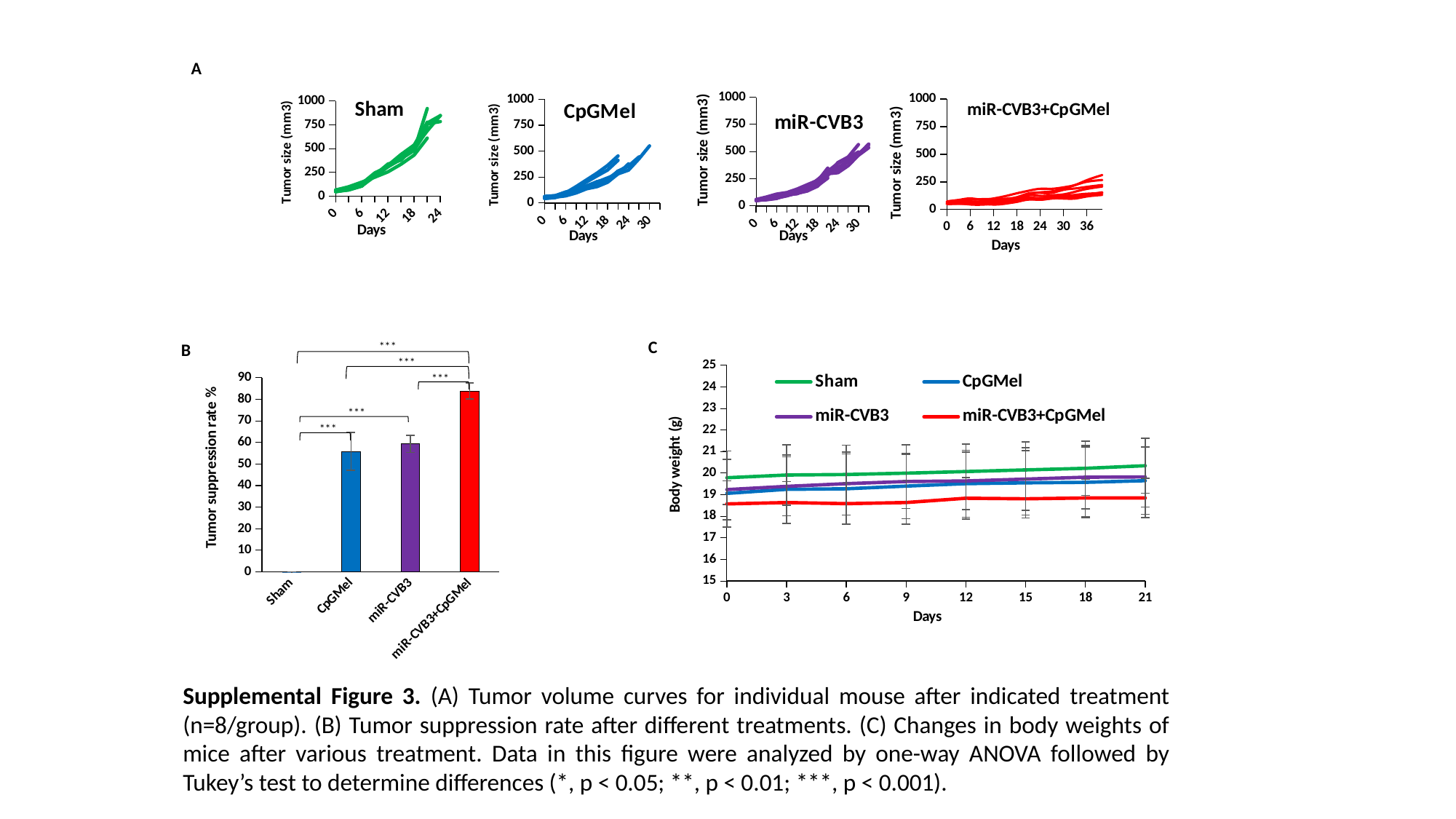

A
[unsupported chart]
[unsupported chart]
[unsupported chart]
### Chart: miR-CVB3+CpGMel
| Category | | | | | | | | |
|---|---|---|---|---|---|---|---|---|
C
B
***
### Chart
| Category | | | | |
|---|---|---|---|---|
| 0 | 19.7875 | 19.0625 | 19.2375 | 18.574999999999996 |
| 3 | 19.9125 | 19.25 | 19.387500000000003 | 18.6375 |
| 6 | 19.9375 | 19.275 | 19.5125 | 18.5875 |
| 9 | 20.0 | 19.4 | 19.6125 | 18.637499999999996 |
| 12 | 20.075 | 19.512500000000003 | 19.637500000000003 | 18.8375 |
| 15 | 20.150000000000002 | 19.549999999999997 | 19.725 | 18.8125 |
| 18 | 20.224999999999998 | 19.575000000000003 | 19.8125 | 18.85 |
| 21 | 20.342857142857145 | 19.650000000000002 | 19.824999999999996 | 18.85 |***
### Chart
| Category | |
|---|---|
| Sham | 0.001 |
| CpGMel | 55.85 |
| miR-CVB3 | 59.34 |
| miR-CVB3+CpGMel | 83.81 |
***
***
***
Supplemental Figure 3. (A) Tumor volume curves for individual mouse after indicated treatment (n=8/group). (B) Tumor suppression rate after different treatments. (C) Changes in body weights of mice after various treatment. Data in this figure were analyzed by one-way ANOVA followed by Tukey’s test to determine differences (*, p < 0.05; **, p < 0.01; ***, p < 0.001).

## Slide 4
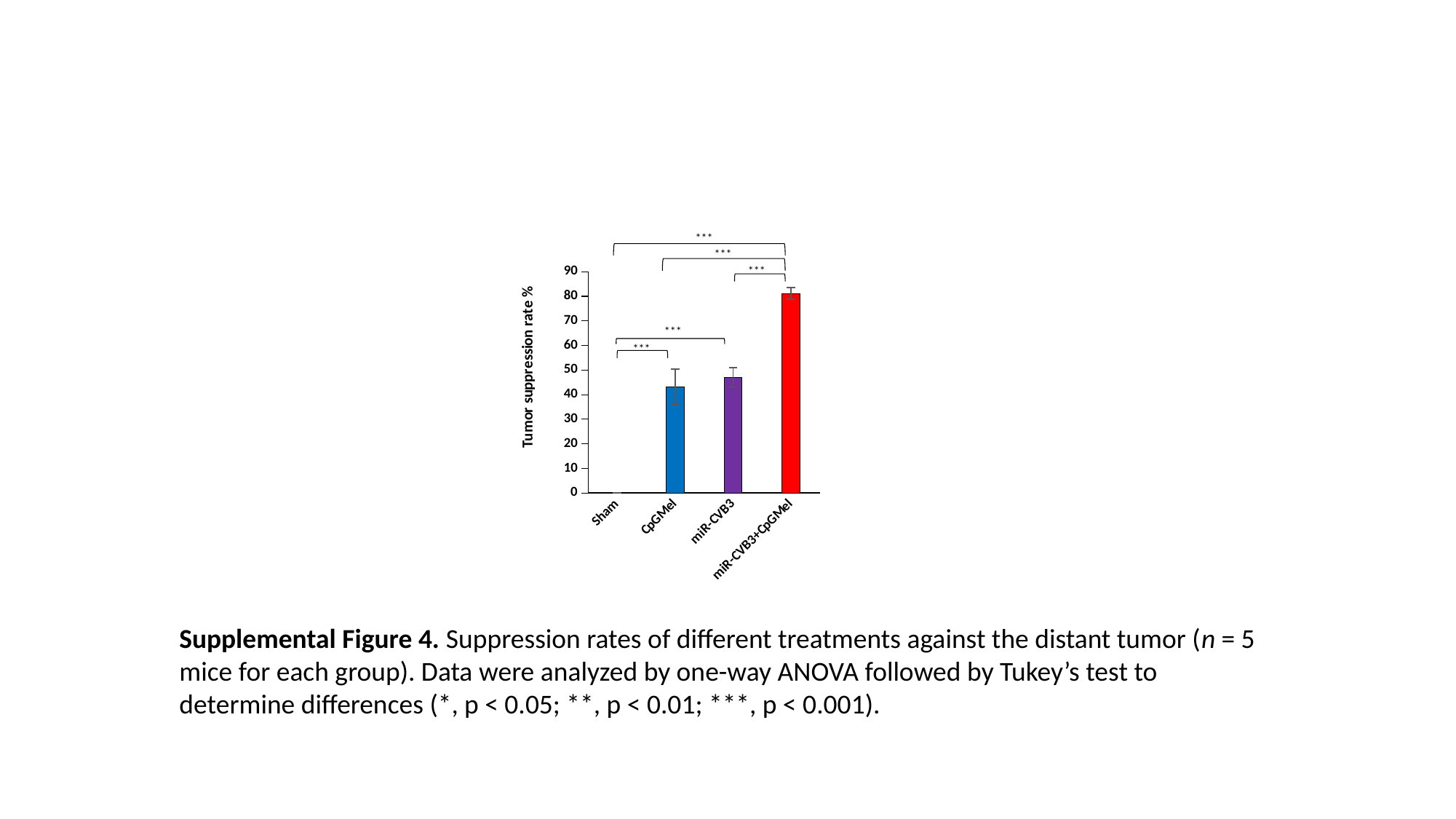

***
***
### Chart
| Category | |
|---|---|
| Sham | 0.001 |
| CpGMel | 43.17 |
| miR-CVB3 | 47.08 |
| miR-CVB3+CpGMel | 81.18 |***
***
***
Supplemental Figure 4. Suppression rates of different treatments against the distant tumor (n = 5 mice for each group). Data were analyzed by one-way ANOVA followed by Tukey’s test to determine differences (*, p < 0.05; **, p < 0.01; ***, p < 0.001).

## Slide 5
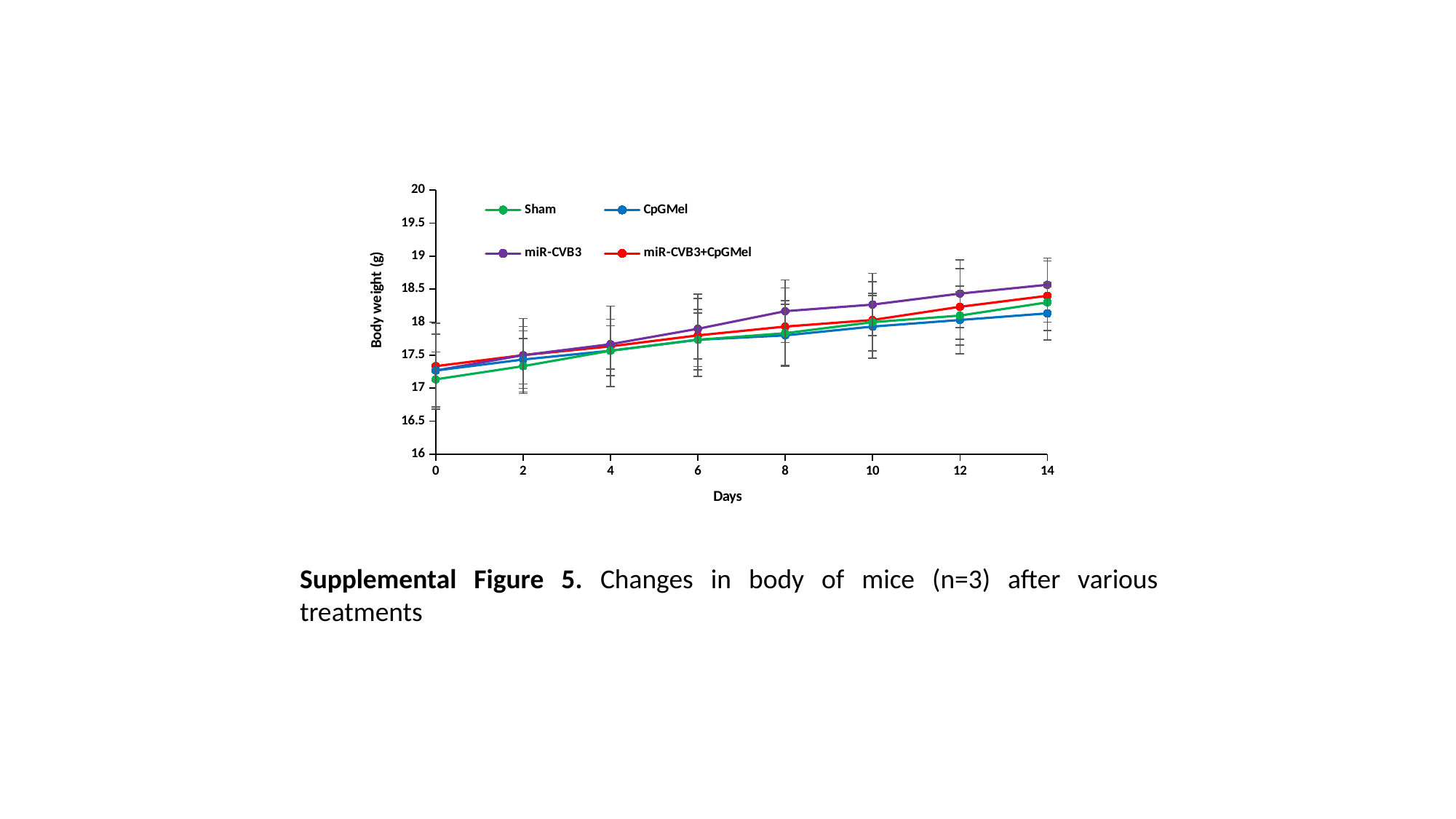

### Chart
| Category | | | | |
|---|---|---|---|---|Supplemental Figure 5. Changes in body of mice (n=3) after various treatments
