# Supplementary material for: A combination of genetically engineered oncolytic virus and melittin-CpG for cancer viro-chemo-immunotherapy
Source: BMC Med. 2023 May 24;21:193. doi: 10.1186/s12916-023-02901-y (PMC10210435; doi:10.1186/s12916-023-02901-y)
Supplement: Supplementary file 2 — Additional file 2. [file 12916_2023_2901_MOESM2_ESM.pptx]

## Slide 1
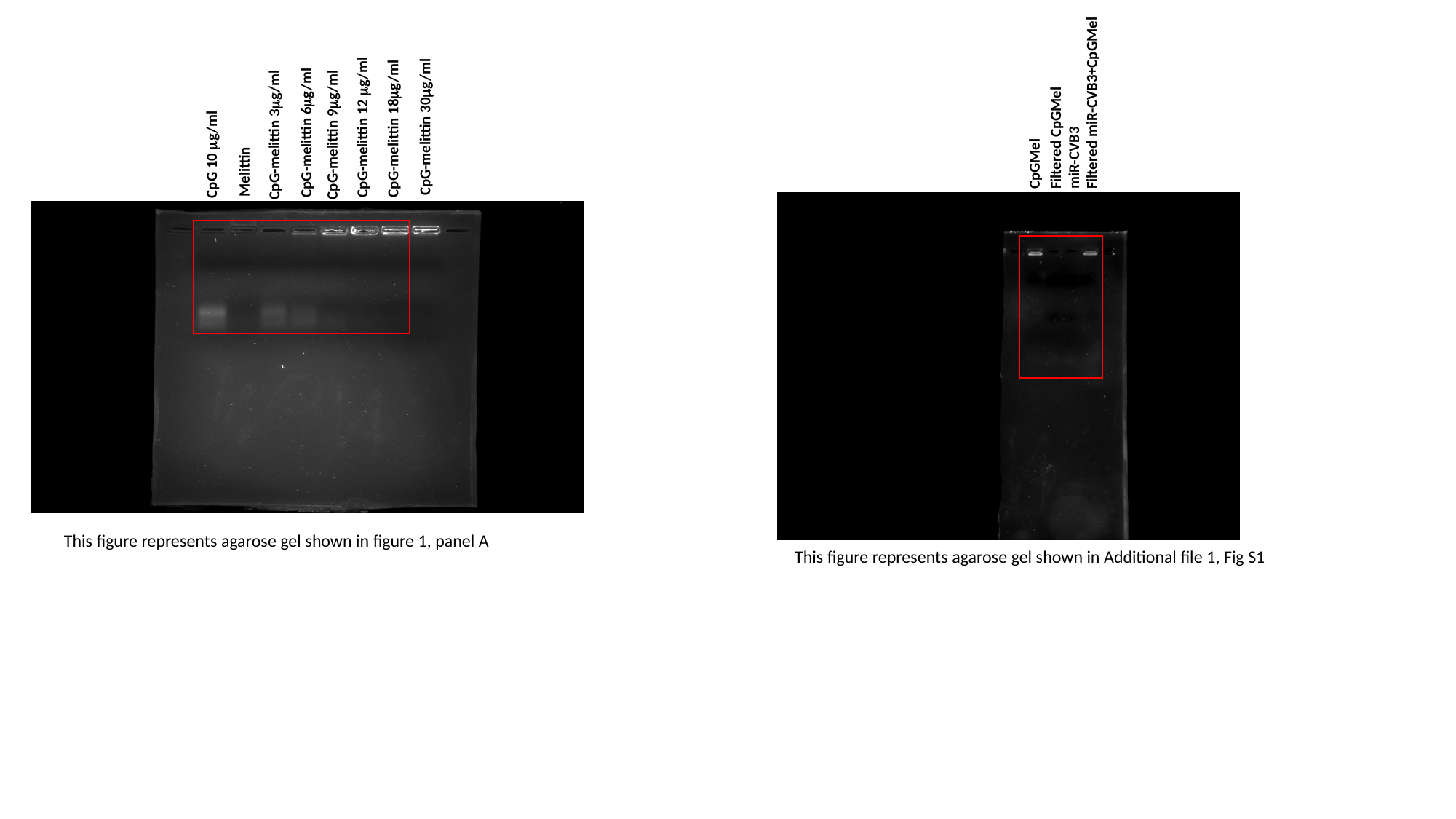

CpG-melittin 12 μg/ml
CpG-melittin 18μg/ml
CpG-melittin 6μg/ml
CpG-melittin 9μg/ml
CpG-melittin 3μg/ml
CpG 10 μg/ml
Melittin
Filtered miR-CVB3+CpGMel
CpG-melittin 30μg/ml
Filtered CpGMel
miR-CVB3
CpGMel
This figure represents agarose gel shown in figure 1, panel A
This figure represents agarose gel shown in Additional file 1, Fig S1

## Slide 2
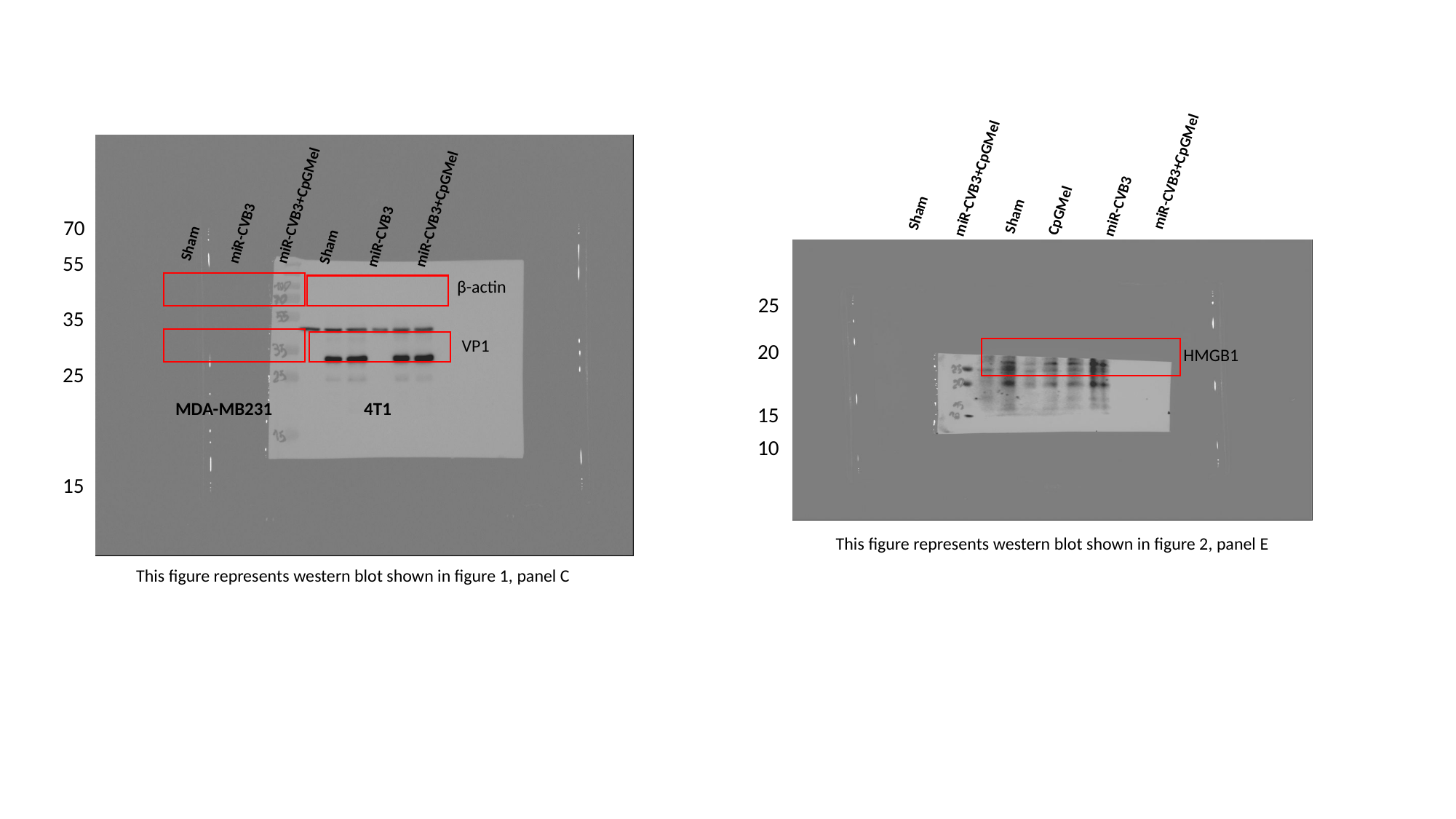

miR-CVB3+CpGMel
miR-CVB3+CpGMel
miR-CVB3+CpGMel
miR-CVB3
miR-CVB3+CpGMel
CpGMel
Sham
Sham
70
miR-CVB3
miR-CVB3
Sham
Sham
55
β-actin
25
35
VP1
20
HMGB1
25
4T1
MDA-MB231
15
10
15
This figure represents western blot shown in figure 2, panel E
This figure represents western blot shown in figure 1, panel C
